# Prevalence of Avoidant/Restrictive Food Intake Disorder in Children Diagnosed with Autism Spectrum Disorder and Their Parents: A Study on the Broad Autism Phenotype

**DOI:** 10.1192/j.eurpsy.2025.495

**Published:** 2025-08-26

**Authors:** B. Ozel, S. Ceran, A. Bağcaz, D. Kaba

**Affiliations:** 1 Department of Psychiatry; 2 Department of Child and Adolescent Psychiatry, Baskent University, Ankara, Türkiye

## Abstract

**Introduction:**

Avoidant/Restrictive Food Intake Disorder (ARFID) was introduced as a diagnostic category in DSM-5 to describe individuals with restrictive eating behaviors without fear of weight gain, differentiating it from Anorexia Nervosa (American Psychiatric Association, DSM-5, 2013). Although prevalent in children with Autism Spectrum Disorder (ASD), ARFID’s co-occurrence with ASD and its prevalence in parents of children with ASD, particularly regarding the Broad Autism Phenotype (BAP), remains underexplored. This study investigates the prevalence of ARFID in both children diagnosed with ASD and their parents, focusing on BAP features in parents and their relationship with ARFID symptoms.

**Objectives:**

1. To determine the prevalence of ARFID in parents of children diagnosed with ASD.
2. To explore the relationship between ARFID symptoms and ASD symptoms in both parents and children, examining the role of BAP in parents.

**Methods:**

A cross-sectional study was conducted at Başkent University Psychiatry Clinic. The sample consisted of 69 children aged 2-18 diagnosed with ASD and 115 parents. ARFID diagnosis was determined using structured clinical interviews and the Nine-Item ARFID Screen (NIAS), while autism symptoms were assessed using the Childhood Autism Rating Scale (CARS) for children and the Autism Spectrum Quotient (AQ) for parents. Psychosocial, anthropometric, and clinical assessments were performed, including body mass index (BMI), nutritional status, and vitamin deficiencies. Statistical analysis included t-tests, chi-square tests, and correlation analyses (SPSS 24).

**Results:**

The study found that 34.8% of children with ASD (n=24) and 13% of parents (n=15) were diagnosed with ARFID, significantly higher than general population estimates (3.5%, p<0.001) (Thomas et al. Curr Psychiatry Rep 2017; 19: 54). Children with ARFID scored higher on the CARS (mean=38.58, SD=7.92) compared to non-ARFID children (mean=33.73, SD=5.59), with a statistically significant difference (t=-2.878, p=0.005). Furthermore, positive correlations were found between ARFID symptoms and autism-related features in both children and parents, particularly in the AQ imagination subscale (r=0.358, p<0.01).

**Conclusions:**

This study highlights a significantly higher prevalence of ARFID in children with ASD and their parents compared to the general population. The strong correlation between ARFID and ASD symptoms, particularly BAP features in parents, suggests shared neurodevelopmental pathways. These findings underscore the need for increased clinical awareness of ARFID in both ASD children and their families, as well as the importance of incorporating ARFID assessments into ASD treatment plans. Future research should focus on the genetic and developmental mechanisms linking ARFID and ASD.

**Disclosure of Interest:**

None Declared

